# Representation of Multidecadal Sahel Rainfall Variability in 20th Century Reanalyses

**DOI:** 10.1038/s41598-018-29217-9

**Published:** 2018-07-19

**Authors:** Ellen Berntell, Qiong Zhang, Léon Chafik, Heiner Körnich

**Affiliations:** 1 Department of Physical Geography and Bolin Centre for Climate Research, Stockholm University, Stockholm, Sweden; 2 Geophysical Institute, University of Bergen, Bjerknes Center for Climate research, Bergen, Norway; 3 Swedish Meteorological and Hydrological Institute (SMHI), Norrköping, Sweden

## Abstract

Summer rainfall in the Sahel region has exhibited strong multidecadal variability during the 20^th^ century causing dramatic human and socio-economic impacts. Studies have suggested that the variability is linked to the Atlantic multidecadal variability; a spatially persistent pattern of warm/cold sea surface temperatures in the North Atlantic. In the last few years, several promising century-long reanalysis datasets have been made available, opening up for further studies into the dynamics inducing the observed low-frequency rainfall variability in Sahel. We find that although three of the 20^th^ century ECMWF reanalyses show clear multidecadal rainfall variability with extended wet and dry periods, the timing of the multidecadal variability in two of these reanalyses is found to exhibit almost anti-phase features for a large part of the 20^th^ century when compared to observations. The best representation of the multidecadal rainfall variability is found in the ECMWF reanalysis that, unlike the other reanalyses (including NOAA’s 20^th^ century), do not assimilate any observations and may well be a critical reason for this mismatch, as discussed herein. This reanalysis, namely ERA-20CM, is thus recommended for future studies on the dynamics driving the multidecadal rainfall variability in Sahel and its linkages to the low-frequency North Atlantic oceanic temperatures.

## Introduction

Multidecadal variability is a global phenomenon of climate variability^1–3^, where the Sahel rainfall is one of the most prominent examples^4,5^. Previous observational data show that Sahel rainfall has exhibited clear, extended dry and wet periods during the 20^th^ century in conjunction with strong inter-annual variability, with severe consequences for the people living in the region as a result^4,5^. The long persistent drought in 1970s and 1980s and later rainfall recovery drew attention to the regime shift in Sahel and emphasized the need for more research into the causes of the decadal climate change^6^. However, identifying the exact driving dynamics behind the low-frequency variability of the Sahel rainfall is challenging. Statistical analysis has indicated that the multidecadal variability in Sahel is associated with periods of warm/cold sea surface temperatures (SSTs) in the North Atlantic, referred to as the Atlantic Multidecadal Variability (AMV)^7–9^, a phenomenon that has been linked to climate variability in several regions globally^7,8,10^. At the same time, the understanding of the dynamics driving the observed multidecadal climate variability in Sahel and the possible dynamical link to the AMV has long been hindered by the lack of century-long, dynamically consistent datasets. Studies suggest that the warm/cold phases of the AMV warms/cools the Sahara region which in turn strengthen/weaken the Saharan Heat Low, an important feature of the West African Monsoon^11–13^. Comparisons of the extended wet and dry periods using reanalysis with shorter time-spans indicate that this in turn strengthens the meridional circulation, the low-level westerlies and the African Easterly Jet, increases the moisture flux convergence in the Sahel and intensifies the West African Monsoon leading to increased rainfall in the region^11,13,14^. As these studies are performed using shorter time-series with often un-filtered data it is however still difficult to separate influence from high- and low-frequency variability.

In the last few years several new reanalysis datasets have been released which span the entire 20^th^ century both using coupled and un-coupled models (e.g. the coupled ECMWF 20^th^ century reanalysis (CERA-20C)^15^. These datasets are currently being used for e.g. studies on dynamical processes and evaluation of models in many different regions and fields globally^16,17^. Due to their long time-coverage it also opens up the opportunity to study low frequency rainfall variability in Sahel. In addition, the complete dynamical framework in reanalysis would certainly broaden and deepen our understanding on the multidecadal variability. But it is, first and foremost, dependent on whether these reanalyses can capture the realistic multidecadal variability of Sahelian rainfall as seen in observations. Furthermore, as some of these reanalyses are driven by the observed Sea Surface Temperature (SST), one can expect the similar variability would appear in the forced atmosphere and climate.

In this study, we examine the representation of the multidecadal rainfall variability in Sahel using four recently released reanalysis datasets; the un-coupled, coupled and AMIP-style European Centre for Medium-Range Weather Forecasts’ (ECMWF) 20^th^ century reanalyses (ERA-20C, CERA-20C and ERA-20CM) and the National Oceanic and Atmospheric Administration (NOAA) and the Cooperative Institute for Research in Environmental Sciences (CIRES) 20^th^ century reanalysis version 2 (20CRv2). The results are compared to the rainfall as described by the observational Climate Research Unit (CRU) TS3.24.01 dataset^18^ in order to quantify how well they represent the multidecadal rainfall variability in Sahel. The datasets represent different methods of producing reanalyses data, and through evaluation and inter-comparison different possible causes for discrepancies are discussed.

The best representation of the Sahelian rainfall is obtained by the ERA-20CM dataset which, surprisingly, in spite of not assimilating any observations, recreate the multidecadal rainfall variability well throughout the 20^th^ century. The ERA-20C and CERA-20C datasets do not realistically reproduce the Multidecadal Sahel rainfall variability, and our results indicate that the assimilation process might play an important role in the clear mismatch of this multidecadal feature. The 20CRv2 dataset exhibits a lower multidecadal variability compared to the ECMWF datasets, making it less suitable for analysis than other available 20^th^ century reanalyses.

## Reanalysis Datasets

The three ECMWF reanalyses are produced using an Integrated Forecast System (IFS) with the horizontal resolution of T159 (Table 1) and the radiative forcing and atmospheric composition is as specified for the Coupled Model Intercomparison Project Phase 5 (CMIP5)^19^. ERA-20C is a single member reanalysis that is forced by sea surface temperatures and sea ice concentrations from the HadISST dataset version 2.1.0.0 control member. The reanalysis assimilates surface pressure and marine wind observations from the International Surface Pressure Databank (ISPD)^20^ version 3.2.6 and the International Comprehensive Ocean-Atmosphere Data Set (ICOADS)^21^ version 2.5.1.

**Table 1:** Characteristics of the four reanalysis datasets used in this study.

Name	Organization	Temporal coverage	Horizontal resolution	Assimilation	SST and sea-ice forcing	Ensemble members	References
ERA-20C	ECMWF	1900–2010	T159 ~ 125 km	4D-Var	HadISST2.1.0.0	—	Poli *et al*. (2015)^25^
CERA-20C	ECMWF	1901–2010	T159 ~ 125 km	4D-Var	Coupled ocean-ice model	10	Laloyaux *et al*. (2016)^14^
ERA-20CM	ECMWF	1900–2010	T159 ~ 125 km	—	HadISST2.1.0.0	10	Hersbach *et al*. (2015)^21^
20CRv2	NOAA/CIRES	1871–2013	T62 ~ 210 km	Ensemble Kalman Filter	HadISST1.1	56	Compo *et al*. (2011)^24^

The ECMWF and NOAA/CIRES reanalyses use different versions of the observations used for assimilation and SST/SIC forcing. HadISST2 has a different land/sea mask and contains more ocean grid-boxes compared to HadISST1. It is also based on monthly mean values rather than the monthly median as is the case for HadISST1. ECMWF also use ISPD v.3.2.6 and ICOADS v.2.5.1 while NOAA/CIRES use ISPD v.2.

The ERA-20CM dataset is an ensemble of 10 atmospheric model realizations. It does not assimilate any atmospheric observations and is thus not strictly a reanalysis dataset, but we will for simplicity refer to it as such in this article. The ensemble members are forced by 10 different realizations of the HadISST2.1 dataset which represent different plausible evolutions of SSTs and sea ice concentration (SIC). The spread of the ensemble reflects the uncertainties of the SST/SIC observations, and decreases throughout the 20^th^ century as the observational density increases^22^.

The CERA-20C is a coupled reanalysis dataset consisting of 10 ensemble members, which uses the coupled CERA system to assimilate surface pressure and marine winds^23^. Ocean-atmosphere feedback is created through coupling of the IFS for the atmosphere, waves and land to the ocean model Nucleus for European Modelling of the Ocean (NEMO), and the air-sea interface is relaxed with a Newtonian relaxation scheme towards SSTs from the HadISST2.1. In production, the reanalysis period (1900–2010) was divided into 14 streams, and the IFS is started with initial conditions from ERA-20C and NEMO from the 10-member ensemble ORA-20C. The ORA-20C members are initialized with different initial conditions, representing probable ocean states for the year 1900^24^.

NOAA/CIRES 20CRv2^25^ is a 56-member ensemble that assimilates surface pressure and sea level pressure observations from ISPD version 2 using an Ensemble Kalman Filter. It is forced by SST and SIC from HadISST1 and only the ensemble mean is presented in this article.

### Sahel Multidecadal rainfall variability in CRU observations

**Figure 1:**
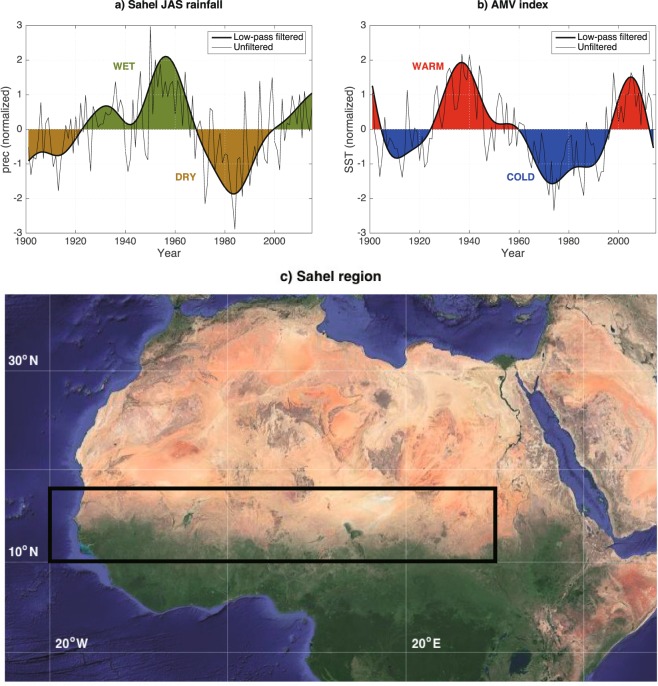
Observed multidecadal Sahel rainfall and Atlantic SST variability. Detrended (**a**) CRU Sahel July-September rainfall 1901–2015, averaged over the domain (20°W-30°E, 10–18°N) and (**b**) HadSST2 annual mean SSTs 1901–2014, averaged over the North Atlantic (75–7.5°W, 0–60°N). Coloured anomalies are low-pass filtered using a Butterworth filter with a 20-year cut-off frequency, thin black lines are unfiltered. All anomalies are normalized by the standard deviation of the corresponding data: 11.4 mm/month rainfall and 0.18 °C for the low-pass filtered Sahel rainfall and AMV index respectively. (**c**) The Sahel region used for the Sahel Rainfall Index indicated in black, Map data: Google, ORION-ME, TerraMetrics.

Given the fact that most annual mean rainfall is contributed by the West African Monsoon season from July to September^4,26^, we define a Sahelian Rainfall Index (SRI) averaged over the domain (20°W–30°E, 10–18°N) for July, August and September (JAS) mean to analyse the variability from 1901 to 2015. The SRI is detrended and low-pass filtered to extract the multidecadal variability. As shown in previous studies^4,7^, the SRI exhibits clear multidecadal variability with dry conditions in the 1900–1920s and 1970–2000s, and wet conditions in the 1920–1960s and 2000 onwards (Fig. 1a). The dominating period of the variability is 60–80 years (see Supplementary Figure S1a), and the wet and dry regimes largely coincide with the periods of warm and cold SSTs in the North Atlantic (Fig. 1b). The AMV index (SST averaged over the domain 75–7.5°W, 0–60°N) exhibits warm conditions in the 1920–1960s and 1990s onward, and cold conditions in the beginning of the 20^th^ century and in the period 1960–1990s. Similar to the SRI, the period of the AMV is ~60–80 years^1,2,27,28^ and the correlation between the SRI and the AMV for the period 1901–2014 is R = 0.51. The correlation peaks at ~12 years lag with a significant correlation of R = 0.67 (significant at 95% confidence level), with the AMV leading the SRI (Supplementary Figure S1b). It is possible that this delayed response is due to the progression of the AMV signal, which originates in the subpolar North Atlantic and is then advected eastward before reaching the North African continent and the Mediterranean forming a horse-shoe SST pattern and thus affecting the rainfall in Sahel^13,29^, but this is beyond the focus of the present work and will be left for future studies.

### Sahel Multidecadal rainfall variability in 20^th^ reanalyses

**Figure 2:**
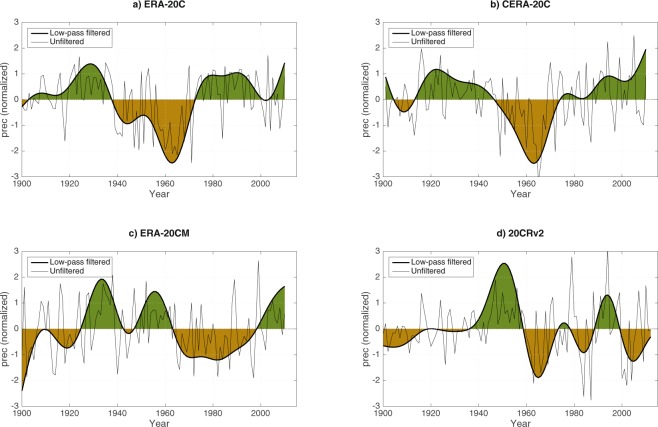
Multidecadal Sahel rainfall variability from reanalyses. Detrended and low-pass filtered Sahelian JAS rainfall for the (**a**) ERA-20C, (**b**) CERA-20C, (**c**) ERA-20CM and (**d**) 20 CRv2 reanalysis datasets. Thin lines are unfiltered, and all anomalies are normalized by the standard deviation of the corresponding data which is 15.6 (ERA-20C), 8.3 (CERA-20C), 6.8 (ERA-20CM) and 7.9 mm/month (20 CRv2) for the low-pass filtered time series.

As shown in Fig. 2, the detrended and low-pass filtered SRI as represented by the ERA-20C, CERA-20C and ERA-20CM reanalyses all exhibit a promising multidecadal variability with a dominating period of 60–80 years (see Supplementary Figure S2a–c). However, the timing of the variability does not match the CRU SRI well and differ from one another. The correlation with the detrended and low-pass filtered CRU SRI for the period 1901–2010 shows a negative correlation for both the ERA-20C and CERA-20C (Table 2). A low correlation coefficient R_LF_ = −0.37 for CERA-20C indicates that the dataset is essentially uncorrelated to the observations. The correlation for the ERA-20C is at R_LF_ = −0.58 (not statistically significant), for the period 1940–1991 the correlation increases to R_LF_ = −0.84 (statistically significant), thereby indicating that for the several decades in the middle of the 20^th^ century the reanalysis is in the opposite phase of the multidecadal variability to the observed ones. For instance, the decades of relatively high Sahelian rainfall seen in observations in the 1940–1960s correspond to a dry regime in the ERA-20C. Among these reanalyses, the ERA-20CM ensemble mean has a high and statistically significant positive correlation to the observation with R_LF_ = 0.77. The correlations for the ten individual members range from R_LF_ = 0.37–0.89 with the highest correlation for ensemble member 3. The 20CRv2 reanalysis is weakly correlated to the observations on the multidecadal scale with R_LF_ = 0.29. Moreover, it appears that although the 20CRv2 reanalysis does contain a variability with a 60–80 years period, the dominating period within this reanalysis’ Sahel rainfall is ~20 years long (Supplementary Figure S2d). The detrended and high-pass filtered reanalysis datasets exhibit positive, statistically significant correlations, *R*_*HF*_, to the observations for all reanalysis datasets, with the highest correlation for the CERA-20C at *R*_*HF*_ = 0.68 (Table 2).

**Table 2:** Correlation (R_LF_: low-pass filtered, R_HF_: high-pass filtered, both detrended) and bias between reanalysis (ERA-20C, CERA-20C, ERA-20CM ensemble mean, NOAA/CIRES 20 CRv2 ensemble mean and ERA-20CM member 3) and CRU observational datasets for the period 1901–2010.

	R_LF_	R_HF_	Bias (mm/month)
ERA-20C	−0.58	**0**.**54**	−51.2
CERA-20C	−0.37	**0**.**68**	−40.3
ERA-20CM	**0**.**77**	**0**.**46**	−28.2
20CRv2	0.29	**0**.**56**	31.4
ERA-20CM e3	**0**.**89**	**0**.**40**	−29.8

Bold font is significantly correlated (>95%), taking the effective degree of freedom into account. Bias is measured in mm/month using Equation 1 on low-pass filtered time series, negative bias indicates dryer conditions compared to observations.

**Figure 3:**
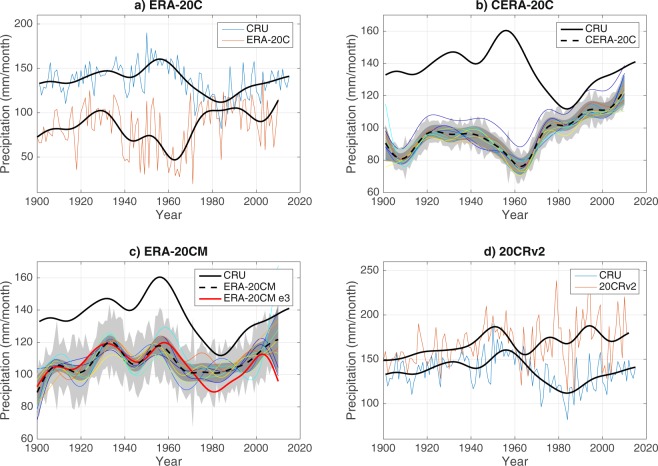
Bias of the observed Sahel rainfall variability in reanalyses. Low-pass filtered Sahelian JAS rainfall (SRI) for CRU and (**a**) ERA-20C, (**b**) CERA-20C, (**c**) ERA-20CM and (**d**) 20 CRv2. Figure 3a,d shows the raw time SRI in addition to the low-pass filtered. Figure 3b,c show the individual ensemble members (coloured lines) together with the ensemble mean (dashed) and standard deviation of the raw (light grey) and low-pass filtered (dark grey) ensemble members. Please note that the x-axis differs for the different subfigures.

Figure 3 shows the low-pass filtered SRI for the four reanalyses datasets together with the observations. Note here the time series are not detrended. It is evident that all ECMWF datasets exhibit a dry bias during the entire 20^th^ century, while the 20CRv2 which instead display elevated levels of rainfall and a wet bias of 31.4 mm/month. The dry bias is largest for the ERA-20C (51.2 mm/month in average) while the CERA-20C and ERA-20CM have lower levels of bias with 40.3 mm/month and 28.2 mm/month respectively.

ERA-20CM ensemble member 3 is, as previously mentioned, the dataset with the single highest correlation to the observations on the multidecadal scale. In Fig. 3c we can see that its multidecadal variability follows that of CRU well over the entire 20^th^ century (the deviation seen for the last decade is a result of the low-pass filtering).

## Discussion

### Overall bias over Sahel in ECMWF reanalyses

The above analysis on the 20^th^ century reanalyses shows that these datasets do exhibit a clear multidecadal rainfall variability in the Sahel region over the past century. However, the dry and wet regimes do not match the observations well and some reanalyses even exhibit an anti-phase relationship. For the absolute rainfall amount, most reanalyses such as ERA20C, CERA-20C and ERA-20CM show a large dry bias. The dry bias feature in ECMWF reanalyses has been reported in previous studies, which show a total column water vapour dry bias in the ERA-20C and ERA-20CM reanalyses^30^. The total column water vapour is resulted from the cloud physics and convection parameterizations, and may therefore be the major reason for the dry bias seen in these reanalyses, not least since they all use the same atmospheric model.

The rainfall in Sahel is highly dependent on the strength and duration of the West African Monsoon whose arrival is caused by development of the Saharan Heat Low in the boreal spring/summer season, and the resulting pressure gradients between the Sahara Desert and the Tropical Atlantic^31,32^. A comparison of the JAS surface temperature over the western Sahara has shown a consistent cold bias in the ERA-20C compared to the observational dataset CRU TS 3.24 by −0.97 K (Supplementary Figure S3). This cold bias could also have led to a reduced rainfall in the Sahel region through a weakening of the West African Monsoon. The cold bias is reduced to −0.69 K in ERA-20CM and the temperature over western Sahara does in CERA-20C exhibit a very weak warm bias of 0.02 K and a stronger warm bias of 0.78 K for 20 CRv2. This is consistent with the dry bias having been reduced for the two ECMWF datasets compared to ERA-20C and 20 CRv2 exhibiting a wet bias, although it is difficult to definitively attribute it to the temperature rather than a systematic dry/wet bias within the models.

The overall improvement seen in CERA-20C compared to ERA-20C evidently indicates that the implementation of a coupled ocean-atmosphere has had a positive effect on the hydrological representation over the Sahel region, even though it does not capture the multidecadal rainfall variability correctly. The dry bias is reduced and the high-frequency variability has a higher correlation to the observations compared to the ERA-20C. As discussed in Laloyaux *et al*.^15^, allowing for realistic and dynamically consistent surface fluxes and feedbacks between the upper ocean and the lower atmosphere ultimately improve descriptions of wind and rainfall. Furthermore, we also note that the multidecadal Sahel rainfall based on the ECMWF reanalyses improve towards the end of the time series compared to observations. In particular, all three datasets exhibit higher correlations and lower dry bias during the satellite era (1979-present) compared to 1901–1975 (pre-satellite era). Although this is somewhat expected, it demonstrates the importance of satellite observations introduced later into HadISST2 dataset in improving the SST forcing, as discussed by Hersbach *et al*.^22^.

### Mismatched timing of the multidecadal variability

**Figure 4:**
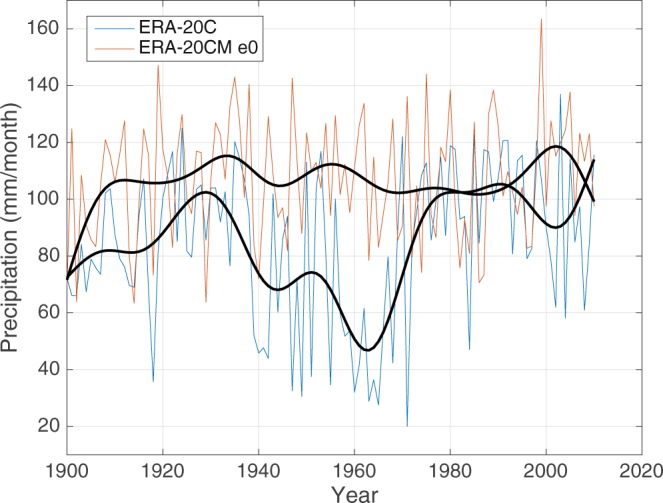
Multidecadal Sahel rainfall in reanalyses with and without assimilation. Detrended and low-pass filtered SRI for ERA-20C (blue) and the ERA-20CM ensemble member 0 (orange) for the period 1900–2010. Both datasets are forced by the same HadISST2 realization and only differ in that ERA-20C assimilates observations while ERA-20CM does not.

The nearly anti-phase feature of multidecadal wet and dry regimes between the CRU and ERA-20C low-pass filtered SRI is a substantial issue, especially for the decades in the middle of the 20^th^ century. Similar anomalies are visible in the CERA20C reanalysis, indicating that the coupling did not improve the timing of the multidecadal variability. Figure 4 shows a comparison between the ERA-20C and the ERA-20CM ensemble member 0 (e0). They are both forced by the same HadISST2 realization and only differs in that ERA-20C includes the assimilation of observations while the ERA-20CM member does not. This is helpful in differentiating the role of the assimilation of observations in the Sahel rainfall. The two time series start with similar levels of rainfall in the year 1900, but they rapidly start to deviate from each other and exhibit distinctly different multidecadal variability throughout the 20^th^ century. The ERA-20CM e0 does not exhibit the same pronounced dry regime in the 1960s which is seen in both the ERA-20C and CERA-20C reanalyses and its multidecadal variability correspond well to the observations, which suggests that the mismatch of the variability pattern could be related to the assimilations. The dry extended regime in ERA20C corresponds to a weakened latitudinal pressure gradient ranging from the 1940s to the end of the 1970s which is not present in the HadSLP2 observational datasets^33^ (Supplementary Figure S4). This further supports the argument that the assimilation may have played a critical role in causing this observed mismatch, as the surface pressure is one of the variables included in the assimilation scheme.

ERA-20C and CERA-20C both exhibit a higher, positive significant correlation to the observations for the high-pass filtered SRI compared to the low-pass filtered. For the ERA-20CM the opposite is true, the reanalysis dataset captures the multidecadal variability to a better extent than the inter-annual variability (see Table 2). This is consistent with previous analysis by Hersbach *et al*.^22^. which found that ERA-20CM outperforms ERA-20C in the representation of global surface temperatures on decadal time scales while ERA-20C is closer to the observations on annual time scales. They suggest that the assimilation of observations in ERA-20C degrades the representation of surface temperature over land on longer time scales while it benefits high frequency, synoptic scale dynamics. Our results indicate that this feature is transferred into the monsoon dynamics over the Sahel region as well, causing the mismatch in rainfall on the multidecadal scale.

The ERA-20CM ensemble members exhibit a large variability throughout the 20^th^ century, with an inter-ensemble variance of 16.6 mm/month in addition to correlation to the observations ranging from R = 0.37 to R = 0.89 on a multidecadal scale. The 10 ensemble members are forced using different SST realizations with distinct but equally plausible SST evolutions in order to take the uncertainty of the observations into account, and the results therefore emphasize the sensitivity of the Sahelian rainfall to the SST forcing. Many studies have indicated the importance of a realistic representation of e.g. North Atlantic, Mediterranean and Tropical Atlantic SST variability for the representation of Sahelian rainfall^34,35^. A comparison of the different HadISST2 realizations used in ERA-20CM would have allowed for further detailed analysis on the sensitivity of the relationship between the SST and rainfall variability, but as they are not yet available therefore this is left for future studies (see notice on https://www.metoffice.gov.uk/hadobs/hadisst2/). This is consistent with our knowledge of the dependence on the land-ocean temperature gradient as well as SST-fields for the monsoon rainfall in Sahel, and underlines the importance of realistic representation of the SSTs. The CERA-20C, which also consists of 10 ensemble members, exhibits an inter-ensemble variance of 8.7 mm/month, i.e. the variance is 48% lower than for ERA-20CM. The ocean model is initialized with different initial ocean states for each ensemble member, but the air-sea interactions are relaxed towards the same HadISST2.1 realization which could have contributed to the reduced variance in CERA-20C.

Compared to the ECMWF-reanalyses, the NOAA/CIRES 20CRv2 dataset exhibits a wet bias of 31.4 mm/month and has a dominating period of ~20 years rather than the 60–80 year period seen in the European reanalyses. The correlation is also lower than ERA-20CM on low-frequency (multidecadal) timescales and lower than CERA-20C on high-frequency timescales (interannual). This makes the dataset less suitable for analysis of the multidecadal Sahel rainfall variability compared to other available century-long reanalysis datasets.

## Conclusion

Multidecadal rainfall variability in the Sahel region still require further research in order to fully understand the governing forcing and dynamical processes. This requires century-long, dynamically consistent datasets in order to better understand the mechanism of the low-frequency variability. We conclude that the high, statistically significant correlation between the ERA-20CM reanalysis and the observational CRU dataset together with the lower dry bias makes this dataset most suitable for low-frequency climate analysis in the Sahel region. As demonstrated herein, the ensemble mean reproduces the multidecadal variability well, but ensemble member 3 has the single highest correlation and is therefore recommended if one attempts to look into the physics inducing the low-frequency Sahel rainfall variability in more detail.

## Method and Data

### Observational datasets

The precipitation was analyzed using high-resolution gridded observational data from the Climate Research Unit (CRU TS 3.24.01)^18^ dataset, consisting of monthly mean values for the years 1901–2015. The dataset is constructed using land-based *in-situ* observations from meteorological stations across the globe, which are interpolated into a grid with 0.5° latitude/longitude resolution. The availability of *in-situ* observations varies in both time and space, and grid cells lacking station data are supplied with their 1961–1990 climatological mean. Approximately 60–80% of the grid cells in Africa have station data for most of the 20^th^ century, with lower coverage at the beginning of the century and for the last decades. The CRU dataset was chosen due to its high spatial resolution and long time series, which is crucial for multidecadal analysis. A comparison to other available century-long observational datasets (e.g. the Global Precipitation Climatology Centre (GPCC)^36^ and the University of Delaware) also showed a high consistency with CRU (Supplementary Figure S5).

Monthly mean sea surface temperatures from the Hadley Centre’s sea surface temperature dataset (HadSST2)^37^ were analyzed for the period 1901–2014.

### Indices

The Sahel rainfall was averaged over the area 10–18°N and 20°W-30°E and over the months July-September to create the Sahel Rainfall Index (SRI) which represents the summer monsoon rainfall in the region. JAS rainfall dominates the annual cycle and represents 73–79% of the mean annual rainfall in both observations and all reanalyses used in this study. To facilitate comparison between the land-only observational dataset and the reanalyses, whose values also cover the ocean, only values over land are used and the ocean is masked out. The AMV index is created in a similar way, by averaging SSTs over the area 0–60°N and 7.5–75°W and calculated as annual mean values. The resulting time series are linearly detrended using a least-square fit and filtered using a Butterworth filter^38^. The low-pass filtering is performed using a 4^th^ order Butterworth low-pass filter with a 20-year cur-off frequency, while the high-pass filtering is performed using a high-pass filter of the same order with a 10-year cut-off frequency.

### Statistical analysis

The correlation is calculated using a Pearson correlation coefficient^39^ for the period 1901–2010, except for the correlation between the CRU SRI and the AMV which is calculated for 1901–2014. The significance of the correlation is calculated taking the effective degree of freedom into account using the Modified Chelton method as described by Pyper and Peterman (1998)^40^. The bias of the reanalysis is calculated on low-pass filtered time series, using Eq. 1, and a power spectrum analysis is performed in order to identify the dominating period of climate variability within the time-series. This is calculated using a Periodogram^41^, and all analysis is performed using Matlab and Climate Data Operators (CDO).

1 $$Bias=\frac{1}{N}{\sum }_{n=1}^{N}(reanalysi{s}_{n}-ob{s}_{n})$$

## Electronic supplementary material

Supplementary Material
