# Supplementary material for: Representation of Multidecadal Sahel Rainfall Variability in 20th Century Reanalyses
Source: Sci Rep. 2018 Jul 19;8:10937. doi: 10.1038/s41598-018-29217-9 (PMC6053360; doi:10.1038/s41598-018-29217-9)
Supplement: Supplementary file 1 — Supplementary Material [file 41598_2018_29217_MOESM1_ESM.pdf]

Representation of Multidecadal Sahel Rainfall Variability in 20th Century Reanalyses

Supplementary Information

Ellen Berntell, Qiong Zhang, Léon Chafik, Heiner Körnich

Supplementary Material

CRU: Power Spectrum Analysis and Lead/Lag correlation of SRI and AMV

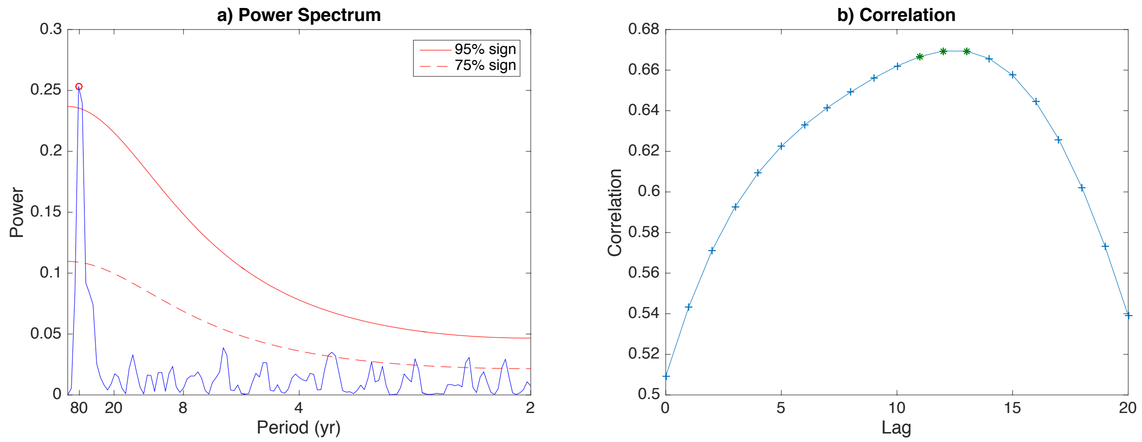

Figure S1: (a) Power spectrum analysis on JAS rainfall in Sahel based on the CRU dataset. Analysis is performed using a Periodogram and shows a dominating period of 60-80 years, indicated in red. The red curves indicate the 95% (solid) and 75% (dashed) confidence level. (b) Lead/Lag correlation between detrended and low-pass filtered CRU SRI and HadSST2 AMV, with the AMV leading the rainfall. The statistically significant correlations (p>95%) are marked in green.

Power Spectrum Analysis of Reanalyses

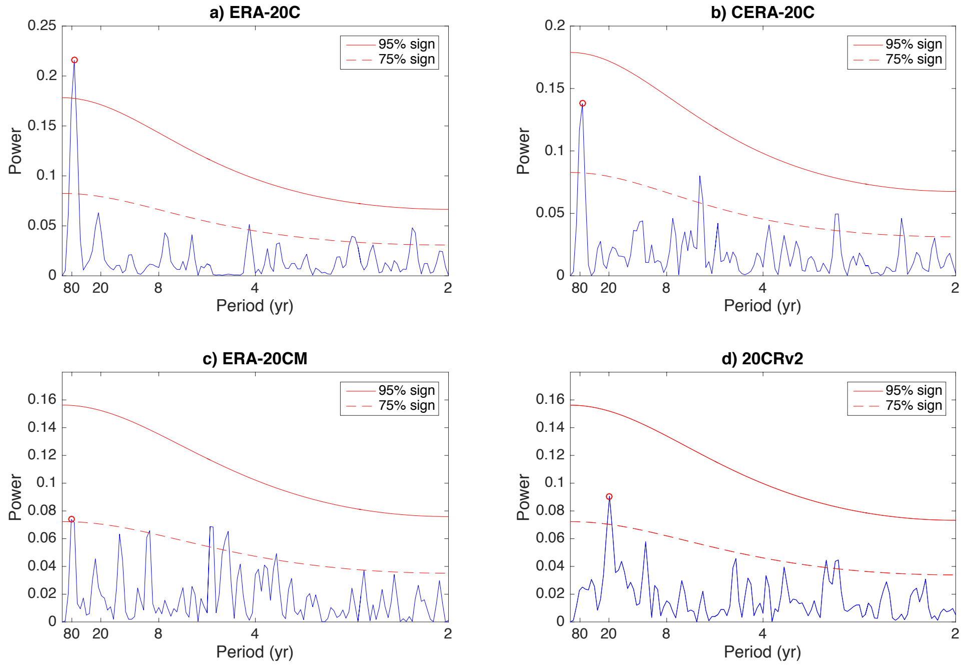

Figure S2: Power spectrum analysis using a Periodogram for a) ERA-20C, b) CERA-20C, c) ERA-20CM and d) 20CRv2 Sahel JAS Rainfall. Dominating periods are indicated in red. ERA-20C, CERA-20C and ERA-20CM exhibit dominating periods of 60-80 years while the dominating period in the 20CRv2 dataset is ~20 years. Red curves show the 95% (solid) and 75% (dashed) confidence level. The multidecadal peaks are not statistically significant at the 95% confidence level for CERA-20C, ERA-20CM and 20CRv2, which is to be expected given the length of the time series. This is discussed in Delworth and Mann (2000) which state that the length of the currently available (non-proxy) observational records of 100-150 years is not enough to enough for definitive multidecadal analysis, but analysis can in spite of this limitation produce interesting insights. The statistical significance was analysed using the method described for Spectral Analysis in the book *Statistical Methods in the Atmospheric Sciences* (Wilks (2011); Equation 8,81). The null-hypothesis is that the peaks in the power spectrum are significantly larger than the red-noise spectrum at that frequency.

Reference:
Wilks, D. S. (2011). Statistical Methods in the Atmospheric Sciences. Academic Press, 3rd edition.

West Sahara temperature bias

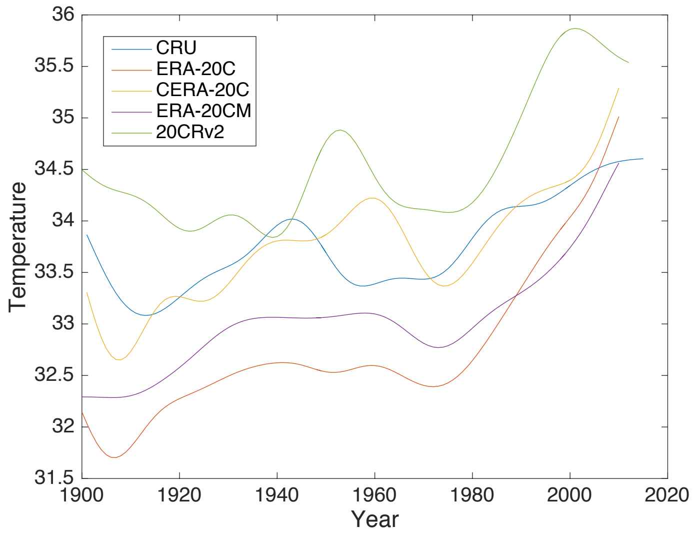

Figure S3: July-September mean CRU TS3.24 near surface temperature (blue) and ERA-20C (orange), CERA-20C (yellow), ERA-20CM (purple) and 20CRv2 (green) 2-m temperature over western Sahara (20-30 ^{o}N, 7.5 ^{o}W – 4 ^{o}E). Bias between the low-pass filtered CRU and reanalyses time series (1901-2010) is -0.97 K for ERA-20C, 0.02 K for CERA-20C, -0.69 K for ERA-20CM and 0.78 K for 20CRv2.

West Africa pressure gradient

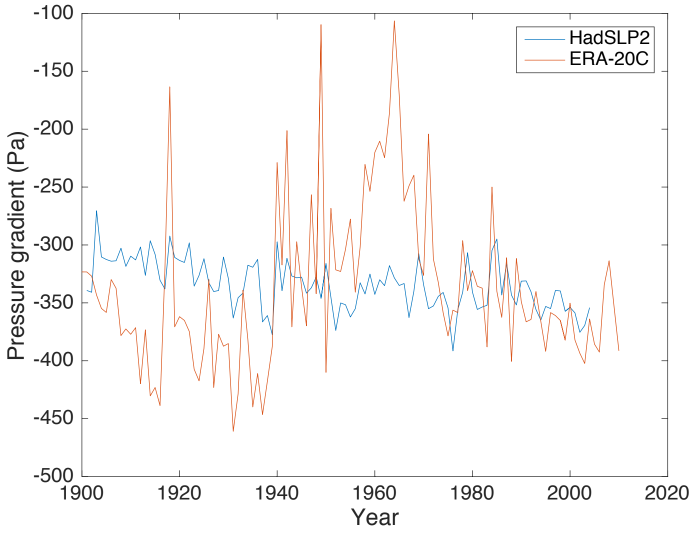

Figure S4: Latitudinal gradient of Sea Level Pressure for HadSLP2 (blue) and ERA-20C (orange).

Gradients are defined as difference between longitudinal averages (10 ^{o}W – 25 ^{o}E) at 7 ^{o}N and 20 ^{o}N.

Sahel rainfall index

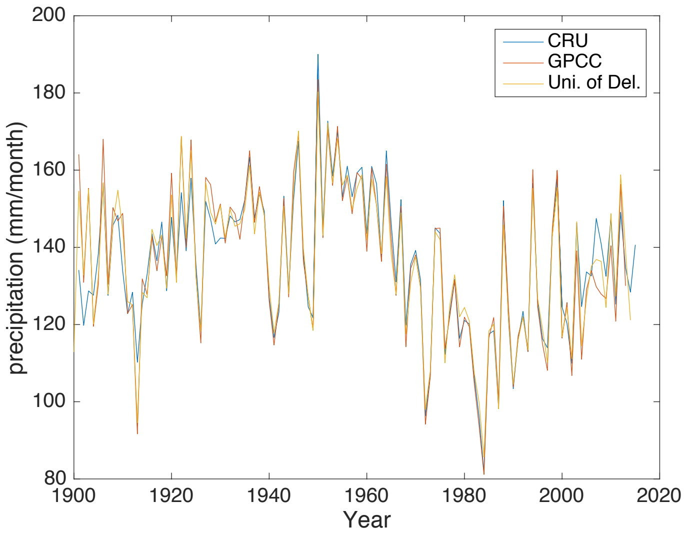

Figure S5: Sahel Rainfall Index (July-September rainfall, averaged over the area 10-18°N and 20°W-30°E) for CRU (blue; 1901-2015), GPCC v7.0 (red; 1901-2013) and University of Delaware v4.01 (yellow; 1900-2014).
